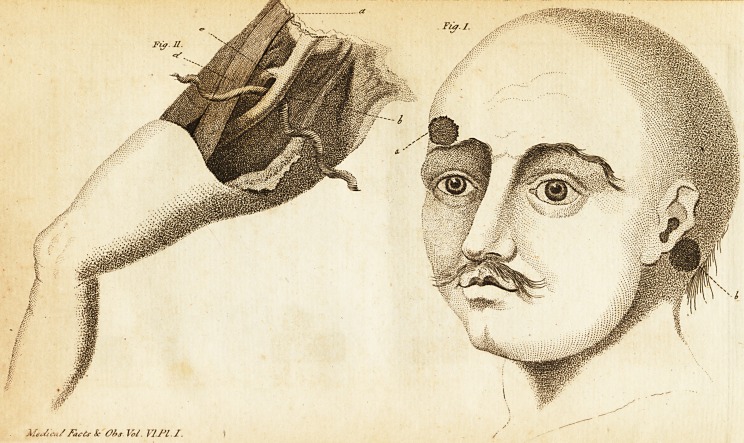# Case of a Gun-Shot Wound of the Head

**Published:** 1795

**Authors:** Henry Yates Carter

**Affiliations:** Surgeon at Kettley, Near Wellington, in Shropshire.


IV. *Caſe of a Gun-Shot Wound of the Head. By the ſame.*

A HESSIAN grenadier, aged between thirty and forty years, being one of a detachment ſent to reduce a fort on the banks of the the Delawar, in the act of levelling his piece, received a ball (grape ſhot) on that part of the os frontis which forms the external canthus of the eye. The ball making its paſſage through the head, came out under and rather behind the oppoſite ear, as in the annexed plate *.

What were the immediate effects upon the receipt of the injury I am not able to ſay, not being immediately upon the ſpot; but he appeared, when brought to the regimental hoſpital, to have a perfect recollection of every circumſtance that had occurred to him, except only for a ſhort time after he fell. He complained of little pain, and did not appear to have loſt ſo much blood as might have been expected.

The ball being a ſpent one, had much ſplintered the cranium, both at its entrance and exit; and was found in the folds of his coat collar.

The wounds being cleanſed, and the ſplinters of bone removed, as far as was practicable, from about the external parts, ſuitable

* See Plate I. Fig. 1. in which *a* refers to the entrance of the ball, and *b* to the part where it paſſed out.

dreſſings

Fig. II.

Fig. I.

dreffings were applied; and his pulfe being full, he was let blood; after which he took twenty-five drops of tincture of opium. The next day he had a fenfe of heavinefs over his eyes, and obferved that objects did not appear to him fo brilliant as ufual; towards the evening he complained of naufea and thirft. He took *tart. vitriol.* and *antim. diaph.* *āā* gr. xii every third hour, and a clyfter was adminiftered. On the third day he complained of pain of his head, accompanied with drowfinefs; and, at intervals, of a weaknefs of his extremities. As the clyfters had failed to procure a fufficient difcharge of fæces, he was directed to take three grains of calomel and fifteen grains of powder of jalap, which operated well, and procured an alleviation of the fymptoms juft now mentioned. His eyes were but flightly inflamed, and he complained of but little pain in that on the affected fide.

On the 6th day there was a good difcharge of matter from the wound, and efcars began to feparate in pretty large floughs. From this time he refted tolerably well without the ufe of the opiate, which till now had been repeated at bed-time. Splinters of bone, that had been driven in at the fuperior wound by the ball, came away away from the dependent orifice at almoſt every dreſſing (which was twice a day) for ſeveral days. The nauſea, head-ach, weakneſs of his limbs, thirſt, and every ſymptom of fever, gradually vaniſhed; the ſuperior orifice filled up with new granulations, and cicatrized firmly; and in about ten weeks there remained nothing more neceſſary than a ſuperficial dreſſing to the inferior opening near the ear.

I did not ſee this man after he had actually left off every application to the affected part; but from the condition of the wound, and the patient's health and vigour, I have not any room to doubt, that in a few days, after I laſt ſaw him, he was capable of returning to his duty.

On reflecting on this extraordinary injury, (inaſmuch as it was not a mortal one) I am inclined to think, that as the ball, though a large one, entered low down upon the orbit, and near the external part of the eye, it miſſed the os planum and frontal ſinuſes, and conſequently that branch of nerves that paſſes through them; ſo that, judging from its apparent direction, it muſt have injured part of the os ethmoides, near the ſeptum naſi. To this courſe of the ball, ball, and the favourable ſituation of the dependent orifice, the favourable event of the caſe was probably owing; for though he complained at certain periods of a ſenſe of weight upon the upper and fore part of the head, general weakneſs of his limbs, and loſs of ſight, ſymptoms indicating an oppreſſion of the brain, yet upon opening the wound, and giving vent to the matter, which was in ſome meaſure confined by the dreſſings, thoſe ſymptoms gradually vaniſhed, and the patient always became perfectly eaſy after the application, for a few minutes, of a warm fomentation.

An inſtance of a ball entering under the right eye, and paſſing obliquely through the cerebrum and cranium above the right ear, without hurting the eye or ſight, is recorded by Heiſter in his Medical, Chirurgical, and Anatomical Caſes and Obſervations, page 7 (of the Engliſh tranſlation) Obſ. VII.

## Figures and Tables

**Fig. I. Fig. II. f1:**